# Characterization of medulloblastoma in Fanconi Anemia: a novel mutation in the BRCA2 gene and SHH molecular subgroup

**DOI:** 10.1186/s40364-015-0038-z

**Published:** 2015-06-06

**Authors:** Evelina Miele, Angela Mastronuzzi, Agnese Po, Andrea Carai, Vincenzo Alfano, Annalisa Serra, Giovanna Stefania Colafati, Luisa Strocchio, Manila Antonelli, Francesca Romana Buttarelli, Massimo Zani, Sergio Ferraro, Amelia Buffone, Alessandra Vacca, Isabella Screpanti, Felice Giangaspero, Giuseppe Giannini, Franco Locatelli, Elisabetta Ferretti

**Affiliations:** Department of Molecular Medicine Sapienza University, Viale Regina Elena 291, 00161 Rome, Italy; Center for Life NanoScience@Sapienza, Istituto Italiano di Tecnologia, Viale Regina Elena 291, 00161 Rome, Italy; Department of Hematology/Oncology and Stem Cell Transplantation, Bambino Gesù Children’s Hospital, IRCCS, Piazza Sant’Onofrio 4, 00165 Rome, Italy; Department of Neuroscience and Neurorehabilitation, Bambino Gesù Children’s Hospital, IRCCS, Piazza Sant’Onofrio 4, 00165 Rome, Italy; Department of Radiology, Bambino Gesù Children’s Hospital, IRCCS, Piazza Sant’Onofrio 4, 00165 Rome, Italy; Department of Radiological, Oncological and Pathological Science, Sapienza University, Viale Regina Elena 291, 00161 Rome, Italy; Department of Experimental Medicine, Sapienza University, Viale Regina Elena 291, 00161 Rome, Italy; Neuromed Institute, Via Atinense 18, 0865 Pozzilli IS, Italy; Pasteur Institute/Cenci Bolognetti Foundation, Viale Regina Elena 291, 00161 Rome, Italy

**Keywords:** Fanconi anemia, Medulloblastoma, *BRCA2*, *FANCD1*, SHH molecular subgroup

## Abstract

Fanconi Anemia (FA) is an inherited disorder characterized by the variable presence of multiple congenital somatic abnormalities, bone marrow failure and cancer susceptibility. Medulloblastoma (MB) has been described only in few cases of FA with biallelic inactivation in the tumor suppressor gene *BRCA2/FANCD1* or its associated gene *PALB2/FANCN.*

We report the case of a patient affected by Fanconi Anemia with Wilms tumor and unusual presentation of two medulloblastomas (MB1 and MB2). We identified a new pathogenetic germline *BRCA2* mutation: c.2944_2944delA. Molecular analysis of MBs allowed us to define new features of MB in FA. MBs were found to belong to the Sonic Hedgehog (SHH) molecular subgroup with some differences between MB1 and MB2. We highlighted that MB in FA could share molecular aspects and hemispheric localization with sporadic adult SHH-MB. Our report provides new findings that shed new light on the genetic and molecular pathogenesis of MB in FA patients with implications in the disease management.

**Electronic supplementary material:**

The online version of this article (doi:10.1186/s40364-015-0038-z) contains supplementary material, which is available to authorized users.

## Background

Fanconi Anemia (FA) is a genetically and phenotypically heterogeneous disorder, inherited with an autosomal (or rarely X-linked) recessive pattern, occurring in approximately 1/100,000 births per year [1]. Main features of FA are the presence of multiple congenital somatic abnormalities, the gradual onset of bone marrow failure, and a strong predisposition to cancer. The most common malignancies include acute myeloid leukemia (AML) and myelodysplastic syndrome (MDS), followed by solid tumors, mostly squamous-cell carcinomas at young age [2–4].

Solid tumors may represent the first manifestation of FA in individuals without congenital somatic abnormalities or hematological manifestations [5]. These tumors include squamous cell carcinomas of head and neck, esophagus, vulva and cervix, liver tumors, and rarely embryonic tumors.

The hallmark of FA is chromosome fragility and hypersensitivity to DNA interstrand cross-linking agents [6, 7, 1, 8–10]. To date, 17 genes (*FANCA*, *FANCB*, *FANCC*, *FANCD1/BRCA2*, *FANCD2*, *FANCE, FANCF, FANCG/XRCC9*, *FANCI*, *FANCJ/BRIP1*, *FANCL*, *FANCM*, *FANCN/PALB2/BRIP1*, *FANCO/RAD51C*, *FANCP/SLX4*, *FANCQ/ERCC4,* and *FANCS/BRCA1*) are known to be involved in the pathogenesis of FA [1, 8, 9, 11]. The 17 gene products appear to interact in a common cellular pathway involved in the regulation of DNA repair. Mutations in eight FA genes (*FANCA, FANCB, FANCC, FANCE, FANCF, FANCG, FANCL*, and *FANCM*) result in loss of *FANCD2* and *FANCI* monoubiquitination, the central regulatory event of the FA pathway, while FA proteins downstream to *FANCD2* (e.g. *FANCJ/BRIP1, FANCN/PALB2,* and *FANCD1/BRCA2*) cooperate in DNA repair [5].

Although a clear picture of genotype-phenotype correlation in FA is currently not completely elucidated, many data suggest that specific complementation groups play a significant role in both phenotypic expression and survival [12, 13].

In particular, biallelic mutations in *FANCD1/BRCA2* (about 2 % of all FA patients) [14] and *FANCN/PALB2* are associated with an extremely high predisposition to leukemia and solid tumors (e.g. medulloblastoma, Wilms tumor) at a very early age [15, 16], with a cumulative incidence probability of malignancy of 97 % by the age of 5.2 years in *FANCD1/BRCA2* patients [17].

The sequential onset of Wilms Tumor (WT), medulloblastoma (MB) and AML has been reported in association with mutation in *BRCA2* [16–32] (Table 1).

**Table 1:** Previously reported cases of brain tumors associated with *BRCA2* mutations

Tumor	Age (yrs)	Sex	*BRCA2* mutation I	*BRCA* mutation 2	Localization	Note	Refs
MB, WT	1.5	F	-	-	NR	-	[22]
MB	3.5	F	c.2830A > T	c.7964A > G	Hemispheric	-	[19, 30]
BT	3	F	7691/insAT	9900/insA	NR	-	[32]
PFT	4.9	M	c.5946delT	9435 T > A	Midline	Ashkenazi Jewish congenital anomalies Sibling of the following	[23, 24]
MAs	2	M	c.5946delT	9435 T > A	Cerebellum	Ashkenazi Jewish Sibling of the previous	[24]
				exon 24			
MB	4.5	F	c.5946delT	c.658_659delGT	Cerebellum	Mixed Ashkenazi Jewish	[24]
MB	2.5	F	5301insA	c.7469 T > C	NR	Latin American	[24]
MB	3.5	F	c.3922G > T	c.9196C > T	NR	African American	[24]
MB	2.3	M	c.658_659delGT	8447 T > A	Hemispheric	Sibling of the following	[16]
MB, WT (15 mo)	4.3	M	c.658_659delGT	8447 T > A	Hemispheric	Sibling of the previous	[16]
MB	2.9	M	-	-	Hemispheric	-	[20]
GB, WT (3.5 yrs)	9	M	c.658_659delGT	c.5645C > A	Cerebellum	Sibling of the following	[27]
MB, WT (7mo) ALL-B (10 yrs)	5.7	M	c.658_659delGT	c.5645C > A	Cerebellum	Sibling of the previous	[27]
PFT	1	F	1548del4	1548del4	Cerebellum	Consanguinity VACTERL syndrome	[25]
MB	3.1	F	c.5946delT	c.9196C > T	Cerebellum	VACTERL syndrome	[17]
PNET or HGG	1.3	M	c.5946delT	c.658_659delGT	Intramedullary	Sibling of the following	[21]
MB	1.7	M	c.5946delT	c.658_659delGT	Hemispheric	Sibling of the previous	[21]
MB	2	NR	c.3264dup	c.IVS19 + 3A > G (c. 8487 + 3A > G)	NR	-	[21]
MB, WT (8 mo) AML (24 mo)	2.9	F	-	-	Hemispheric	Consanguinity	[18, 31]
MB	13	M	c.1114A > C	c.1114A > C	Cerebellum	Desmoplastic histology	[26]
MB WT (4 yrs)	6	F	-	-	Hemispheric	Consanguinity	[28]
MB WT(15 mo)	2.9	F	c.658_659delGT	c.2944delA	MB1: Hemispheric; MB2: vermian	MB1, MB2: SHH subtype	Current report

*MB* Medulloblastoma, *WT* Wilms Tumor, *BT* Brain Tumor, *PFT* Posterior Fossa Tumor, *MAs* Multiple Astrocitomas, *GB* Glioblastoma, *ALL-B* Acute Lymphatic Leukemia – B, *PNET* Primitive Neuroectodermal Tumor, *HGG* High Grade Glioma, *AML* Acute Myeloid Leukemia, *NR* not reported, *yrs* Years, *mo* Months

Here, we report a case of a patient affected by FA, who sequentially developed WT and unexpected two distinct MBs, in which we identified a novel pathogenetic germline *BRCA2* mutation and MB molecular subgroup.

## Case presentation

### Clinical and neurophatologic features

A 15-month-old female patient born at term, small for gestational age, the second daughter of non-consanguineous parents was referred to the Bambino Gesù Children Hospital for macroscopic hematuria due to a large renal neoplasm in a solitary pelvic cake kidney.

Laboratory tests showed mild renal failure (creatinine 1.37 mg/dl, BUN 46 mg/dl), normocytic anemia (Hb 7.5 g/dl; MCV 77 fl), hyperuricemia (8.4 mg/dl) and increased LDH (1963 UI/dl). Hypertension (both diastolic and systolic blood pressure exceeding the 90^th^ percentile) required pharmacological treatment.

Physical examination revealed growth retardation (both weight and height lower than 3^rd^ percentile), abnormal skin pigmentation (both *café au lait* spots and hypopigmentation), elfin facies with epicanthus. Echocardiography showed a mitral valve insufficiency. The patient also had right-convex scoliosis and ribs anomalies. A clinical suspect of FA was formulated and a DEB-test was performed which showed multiple spontaneous and DEB-induced chromosomal breaks per cell. Radiologic tumor assessment confirmed the presence of a renal neoplasm (78.1 × 42.2 × 62.8 mm) (Additional file 1: Figure S1a) with bilateral lung metastases (Additional file 1: Figure S1b).

Neoadjuvant chemotherapy was started, according to SIOP WT 2001 protocol, obtaining a partial response on both primary tumor (44 × 33 × 22 mm) (Additional file 1: Figure S1c) and metastatic lesions. Despite dose-adapted chemotherapy, chosen considering chromosomal fragility and according to renal function, the child experienced a grade-IV hematological toxicity and respiratory failure requiring admission to the intensive care unit. Afterwards, tumorectomy was performed, sparing residual renal functioning tissue. Histologic analysis confirmed a grade-III nephroblastoma, according to SIOP 2001 classification [33]. Post-surgical chemotherapy was resumed until completion of the protocol. At that point, a single residual lung metastasis was present and therefore resected by thoracotomy with no significant complications. Follow up confirmed stable remission with a mild chronic renal failure.

**Fig. 1:**
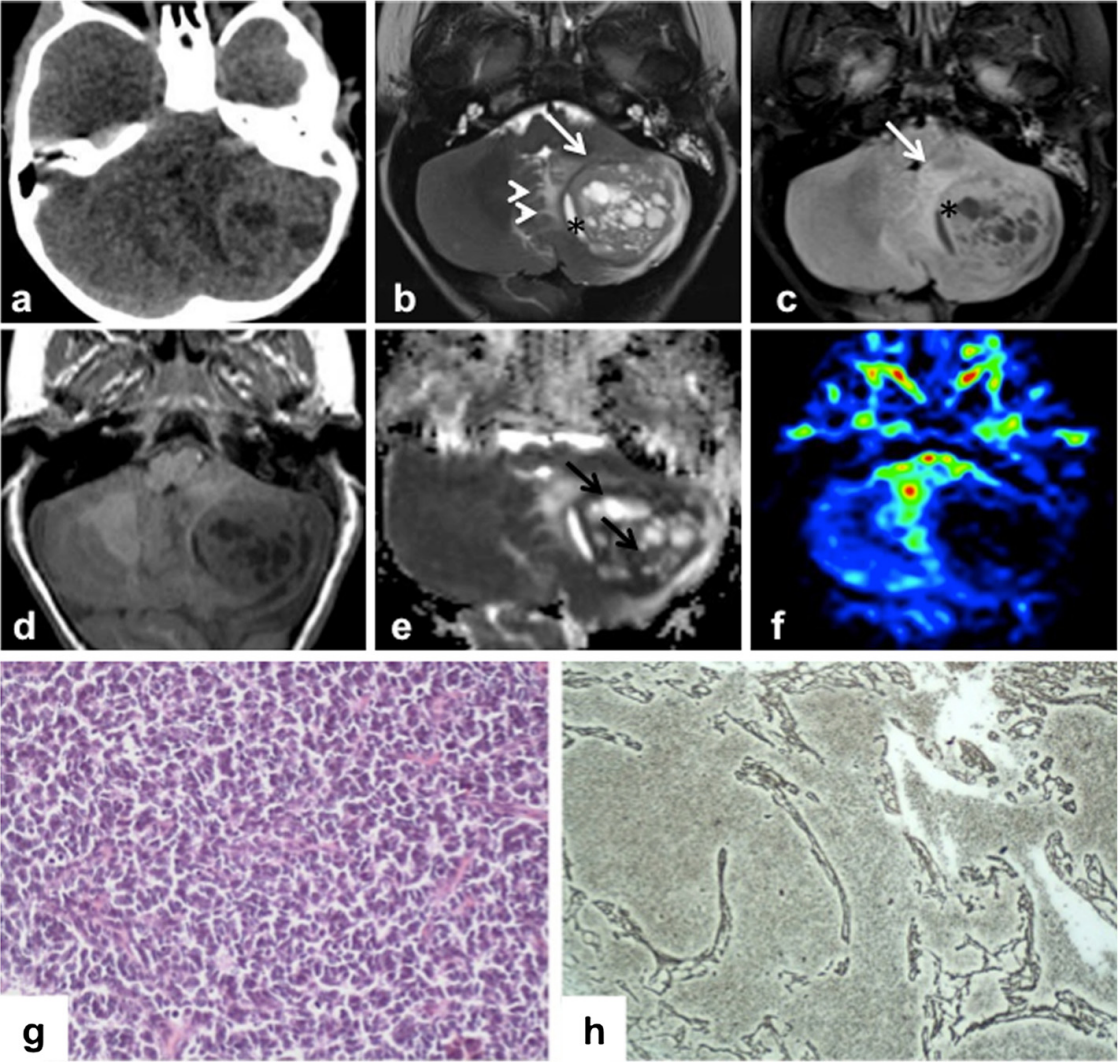
Imaging and Histopatological features of MB1. Axial CT (**a**) and axial conventional MR (**b** T2w, **c** FLAIR T2w, **d** T1w) images demonstrate a heterogeneous posterior fossa tumor in the left cerebellar hemisphere with mild surrounding edema (arrowheads) and significant mass effect on the fourth ventricle. The lesion shows a hypointense rim (white arrows) and a cystic component on its medial side (*). Apparent diffusion coefficient map (**e**) reveals restricted diffusion in solid tissue (black arrows). On the perfusion sequences (**f**) by arterial spin labeling, the tumor was depicted as a low perfusion area. (**g**) Neoplasm is composed of densely cells with round to oval shaped hyperchromatic nuclei with scanty cytoplasm. (**h**) Neoplastic cells are arranged in nodule surrounded by a reticular fiber network (Gomori stain)

At the age of 35 months the child was seen with headache and vomiting at the emergency room. A brain computed tomography (CT) scan showed a left cerebellar hemispheric lesion exerting significant mass effect on the fourth ventricle. Cranio-spinal magnetic resonance imaging (MRI) confirmed a localized rounded heterogeneously enhancing mass with intralesional cystic components and perilesional edema (Fig. 1a-f). Cytologic evaluation of the cerebrospinal fluid resulted negative for neoplastic cells. Gross total resection of the lesion was performed.

Histology showed a neoplasm with an expanded lobular architecture, due to the reticulin-free zones rich in neuropil-like tissue. Such zones contained a population of small cells with rounded nuclei diagnostic of a desmoplastic/nodular medulloblastoma (Fig. 1g-h) without C/N-myc amplification evaluated by FISH analysis. Due to previous clinical history and to the persistence of a mild chronic renal failure, a tailored chemotherapeutic regimen with Carboplatin and Vincristine was chosen. Despite the low dose of chemotherapy employed, the patient presented a significant toxicity after the first course of treatment including pancytopenia (WHO grade IV), sepsis and deterioration of renal function that slowly recovered in 3 months. Considering this relevant toxicity no further treatment was offered.

**Fig. 2:**
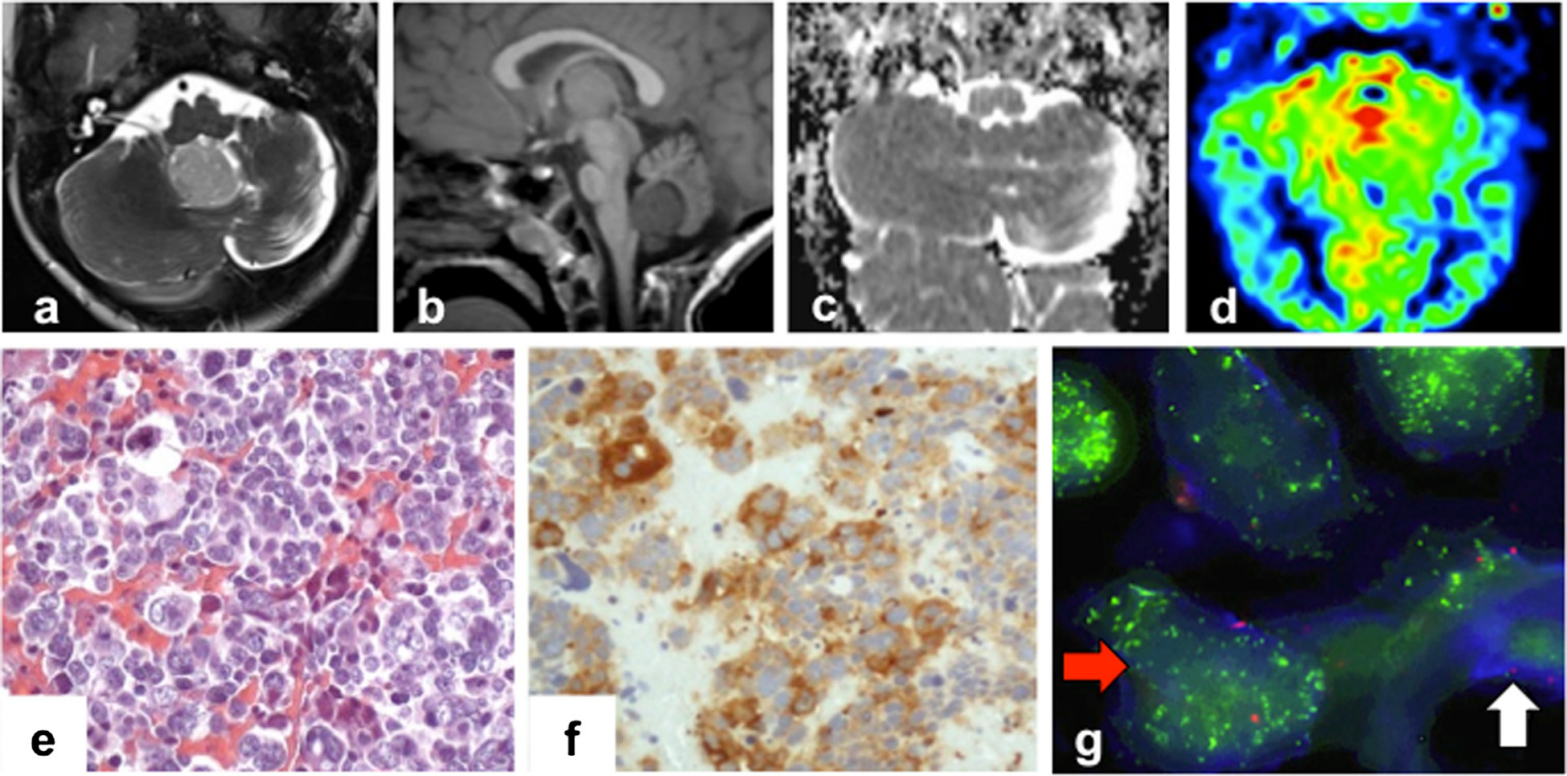
Imaging and Histopatological features of MB2. MRI. Axial T2-weighted image (**a**) and sagittal T1-weighted image (**b**) show a nodular mass originating from the vermis and bulging into the fourth ventricle. Apparent diffusion coefficient (ADC) map (**c**) shows lack of diffusion restriction. Perfusion weighted imaging (**d**) reveals hyperperfusion within the lesion. (**e**) Tumor is characterized by pleomorphic cells with large nuclei, prominent nucleoli and moderate eosinophilic cytoplasm. (**f**) Large cells show immunoreactivity for synaptophysin. (**g**) N-Myc oncogene amplification (green spots) detected in neoplastic nuclei (blue) and centromere 8 signals (red spots) using CEP8/BAC as FISH probes. Red arrow: N-Myc amplified cell. White arrow: N-Myc dyploid cell

At a follow-up MRI, 17 months after the previous surgery for MB, a midline cerebellar mass was detected (Fig. 2a-d). Complete surgical resection was performed, and histology showed a large cell neoplasm with vesicular nuclei, prominent nucleoli and variably abundant eosinophilic cytoplasm consistent with large cell/anaplastic MB (Fig. 2e-f). FISH analysis showed N-myc amplified medulloblastoma cells (Fig. 2g). One month after surgery, a local recurrence with leptomeningeal dissemination was documented.

Considering the toxicity shown during previous treatments, an evaluation of target molecular expression on the tumor was performed. Based on the evidence of expression of P-mTor, a personalized treatment with rapamycin [34] associated with intrathecal liposomal cytarabine [35] was started. Despite therapy, patient died of relapse and disease progression two months after the diagnosis of the second MB at 55 months of age.

### Genetics

Based on published data, the onset of MB after a WT in this FA patient raised the suspicion of a biallelic mutation in *FANCD1/BRCA2*. To confirm this hypothesis, we performed a genetic analysis of *FANC1/BRCA2* on patient’s DNA extracted from both from peripheral blood and MB1 cells, which revealed the presence of compound heterozygosis for *BRCA2* frameshift mutations.

**Fig. 3:**
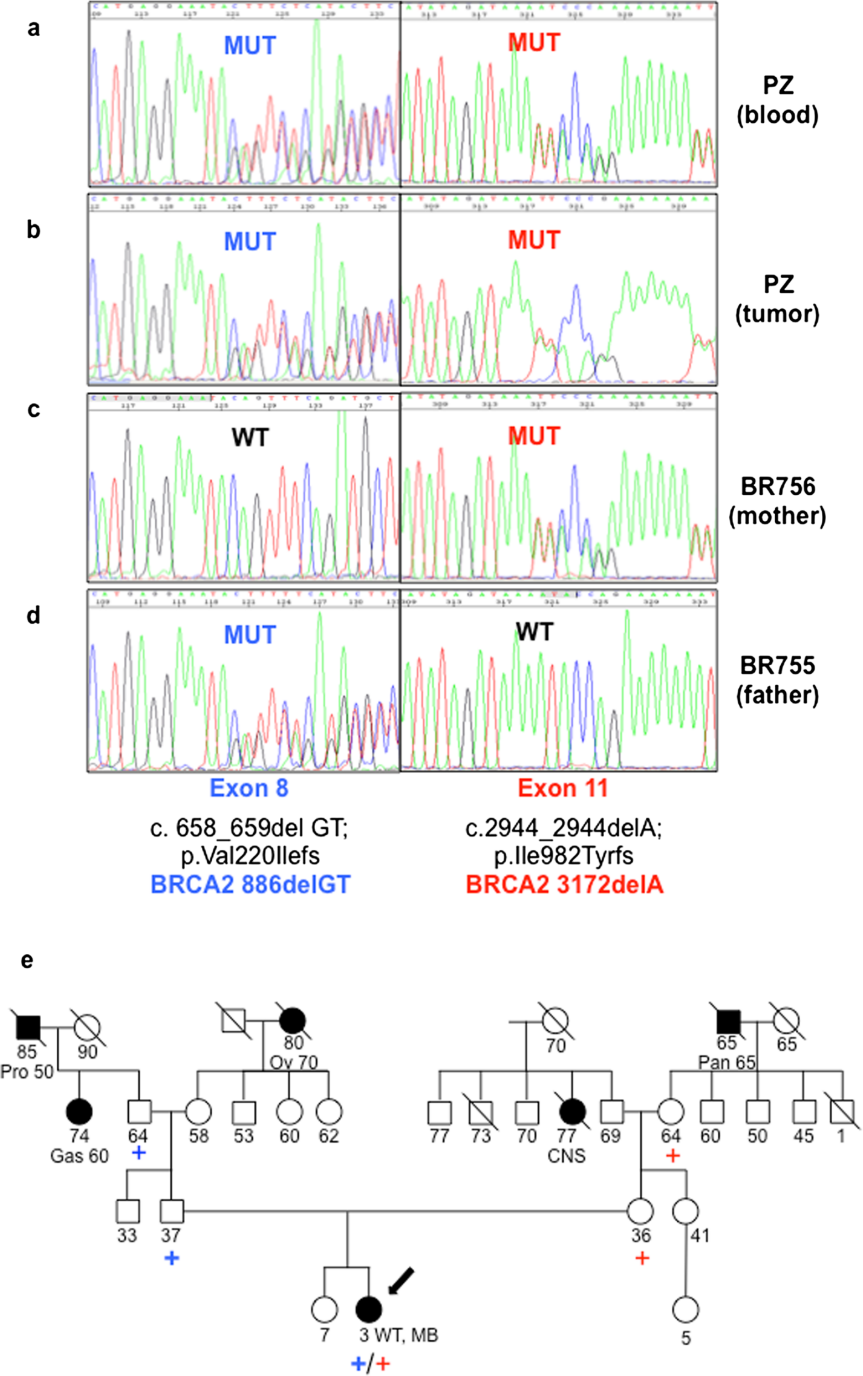
Genetic Analyses. Direct sequencing data relative to the indicated region of *BRCA2* gene demonstrating the biallelic mutations c.2944_2944delA exon 11 and c.658_659delGT exon 8 in patient’s blood (**a**) and tumor (**b**), present in mother’s blood (**c**) and father’s blood (**d**) respectively. **MUT**: mutated case, **WT**: wild type case. (**e**) Family pedigree: closed symbol: affected with cancer, open symbol: unaffected with cancer. The type of cancer and age at presentation are given under the symbol. Arrow: proband, blue plus sign: *BRCA2* c.658_659delGT exon 8 mutation, red plus sign: *BRCA2* c.2944_2944delA exon11 mutation. **Pro**: prostate cancer, **Pan**: pancreatic cancer, **Ov:** ovarian cancer, **CNS**: central nervous system tumor, **Gas:** Gastric cancer, **WT:** Wilms Tumor, **MB:** Medulloblastoma

Gene-specific *BRCA2* analysis, including sequencing of all translated exons and adjacent intronic regions of the *BRCA2* gene, was performed on the DNA samples. Both the truncating and novel genetic variants were confirmed by sequencing two different blood samples on both DNA strands. Sequencing was performed using an ABI PRISM DyeDeoxy Terminator Cycle Sequencing Kit and an ABI 3100 Genetic Analyzer (Thermo Fisher Scientific, Waltham, MA USA). Reference sequence for BRCA2: Genebank, NM_000059.1. By this mean, we revealed a compound heterozigosity for two distinct framshift mutations: the already described c.658_659delGT (p.Val220Ilefs) mutation on *BRCA2* exon 8, and a previously unknown c.2944_2944delA (p.Ile982Tyrfs) mutation on *BRCA2* exon 11 (Fig. 3)a and b, both of which are predicted to lead to truncated BRCA2 proteins. Genetic testing of the parents confirmed they both were heterozygous carriers; in particular, the father carried the c.658_659delGT mutation, while the mother carried the c.2944_2944delA mutation (Fig. 3c and d). Further analysis of the pedigree suggested the segregation of the mutations in the paternal grandfather and in the maternal grandmother, not affected by cancer. The original donor of the new mutation could be the mother’s maternal grandfather affected with pancreatic cancer, which belongs to the spectrum of FA/BRCA-associated tumors (Fig. 3e). Overall, the genealogical chart was characterized by a low occurrence of tumors both in paternal and maternal families and in particular by the absence of breast and ovarian cancer cases (Fig. 3e). Analysis of the MB1 tumor tissue from the affected child revealed heterozygosis for both alleles, thus suggesting both of them are selected for in tumor development.

### MBs molecular features

We performed gene expression analysis of both hemispheric MB1 and vermian MB2 by real-time quantitative PCR (RT-QPCR). We used TaqMan Low Density Array custom designed with TaqMan assays (Life Technologies - Thermo Fisher Scientific, Waltham, MA USA) for genes of interest for medulloblastoma subgrouping, according to Northcott 2012 [36, 37], while we used single TaqMan assay (Life Technologies - Thermo Fisher Scientific, Waltham, MA USA) for all other shown mRNA analyses [38]. Gene expression analysis on samples was performed using a ViiA7 sequence detection system according to the manufacturer’s instructions. RNA of normal human cerebellum (7 adult samples from 25- to 70-year-old subjects were purchased from Biocat (Heidelberg, Germany), Ambion (Life Technologies - Thermo Fisher Scientific, Waltham, MA USA) and BD Biosciences (San Jose, CA) [38].

Unsupervised clustering and heatmaps were generated using the analyzed transcript levels expressed as Delta Ct values as input. We used Spotfire software (TIBCO Software, Inc. CA, USA) to cluster the samples and to generate the heatmaps, as previously described [39, 40].

**Fig. 4:**
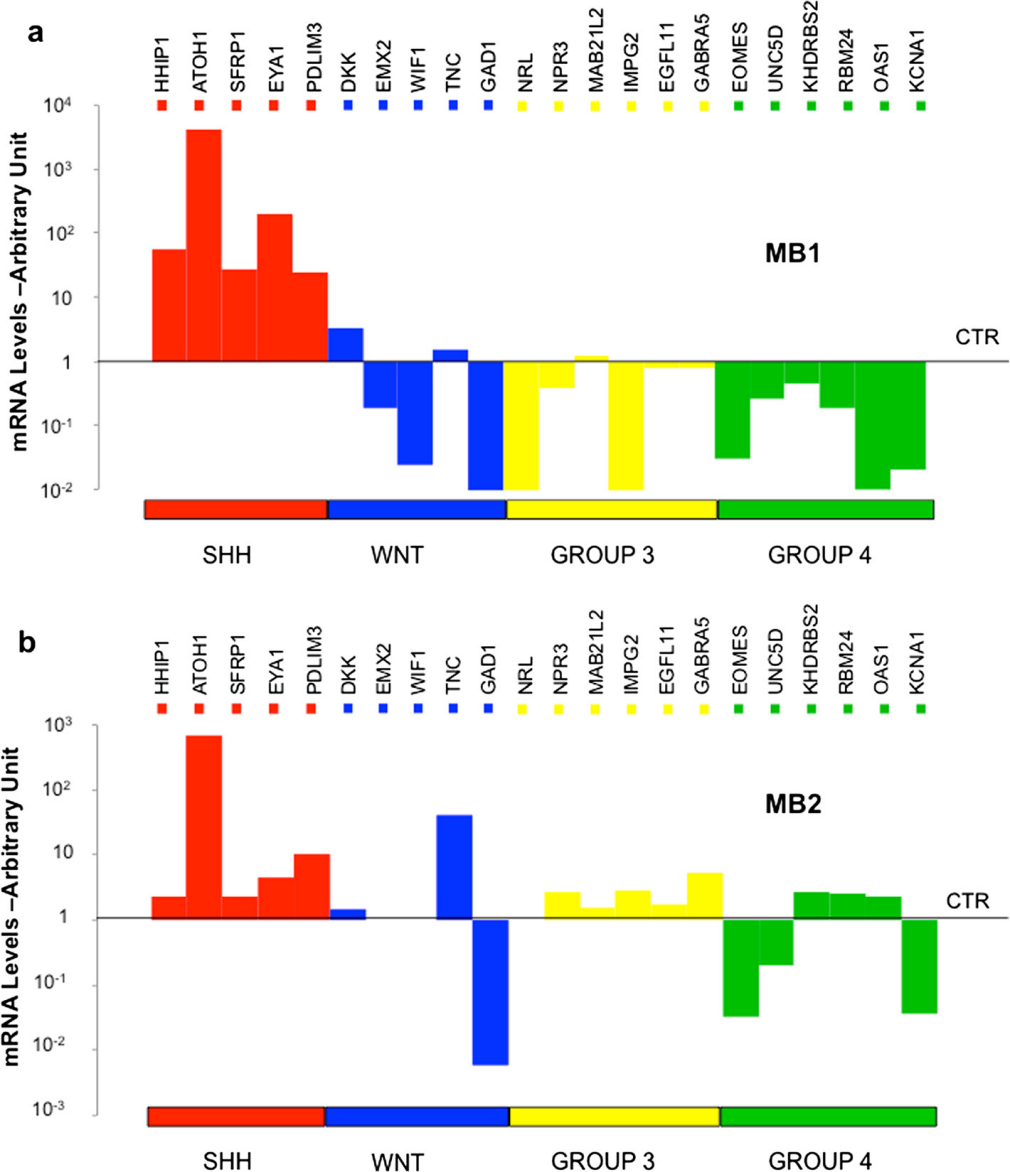
Molecular characterization of MB1 and MB2. Histograms showing mRNA levels of the indicated genes in MB1 (**a**) and MB2 (**b**) compared to normal cerebella (average of *n* = 7) as control (CTR). Genes are grouped and depicted in different colors, depending on the molecular subgroups, which they identify (SHH, WNT, GROUP 3, GROUP 4). The values of Relative Quantification are expressed in log10 scale

The results showed a clear *Sonic-hedgehog (SHH)* molecular subgroup for MB1 (Fig. 4a and Additional file 2: Figure S2) while MB2, though characterized as SHH-MB, was found to express genes associated with group 3 and 4 MBs (such as *GABRA5*, *KHDRBS2* - Fig. 4b).

**Fig. 5:**
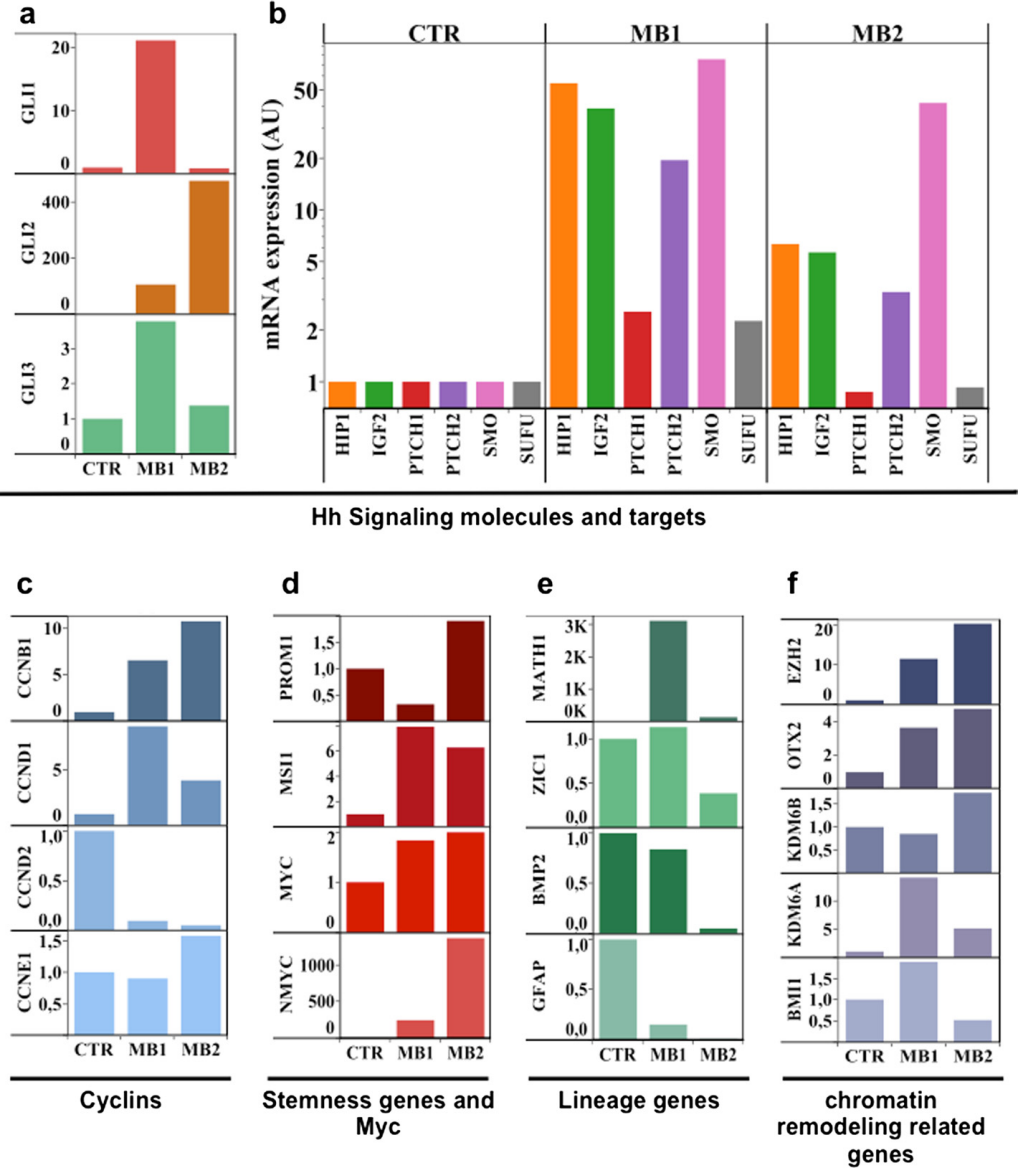
Gene expression analysis of MB1 and MB2. Histograms show mRNA levels of the indicated genes in MB1 and MB2 compared to normal cerebella (average of *n* = 7) as control (CTR). In detail (**a**) GLI family members; (**b**) Sonic Hedgehog pathway (Hh) molecules and direct targets. (**c**) Cyclins. (**d**) Myc genes and Stemness molecules; (**e**) Differentiation molecules; (**f**) Epigenetic modifiers. The values of Relative Quantification are expressed in linear scale for panels (**a**), (**c**), (**d**), (**e**) and (**f**) and log scale for panel (**b**)

Further analysis showed gene expression differences between the two MBs. *Gli1*, the transcription factor of the Hedgehog (Hh) pathway, was highly expressed in MB1, together with all its downstream targets (*PTCH1, PTCH2, IGF2, HHIP, SFRP1, CCND1*) (Fig. 5a and b); while MB2 showed lower levels of *Gli1* and its target genes (Fig. 5b). Conversely, *Gli2* was highly expressed in MB2 (Fig. 5a), while *CCND2* was expressed at very low levels (Fig. 5c). Of note, N-myc amplification was only found in MB2 (Fig. 2g and Fig. 5d). Moreover, differences were found in the expression of stem cell/differentiation genes: MB2 was characterized by low expression of lineage differentiation genes (*ZIC1, BMP2, GFAP, GABRA6*) (Fig. 5e) accompanied by expression of stemness genes, such as *PROM1* and *C-MYC* (Fig. 5d), when compared to MB1.

Genes associated with chromatin modifications, such as histone methylases/demethylases and polycomb (*EZH2, KDM6B, KDM6A, BMI1*) and the homeobox transcription factor OTX2 [41], were also evaluated. Indeed, large cell/anaplastic MB2 showed higher levels of *EZH2*, *OTX2* and *KDM6B* and lower levels of *KDM6A* and *BMI1* (Fig. 5f).

Finally, hemispheric localization of MB is rare in infancy, while is common in adulthood. Moreover, despite the common activation of Hh signaling, infant, childhood and adult SHH MBs have been demonstrated to be clinically, transcriptionally, genetically and prognostically distinct [42, 43].

**Fig. 6:**
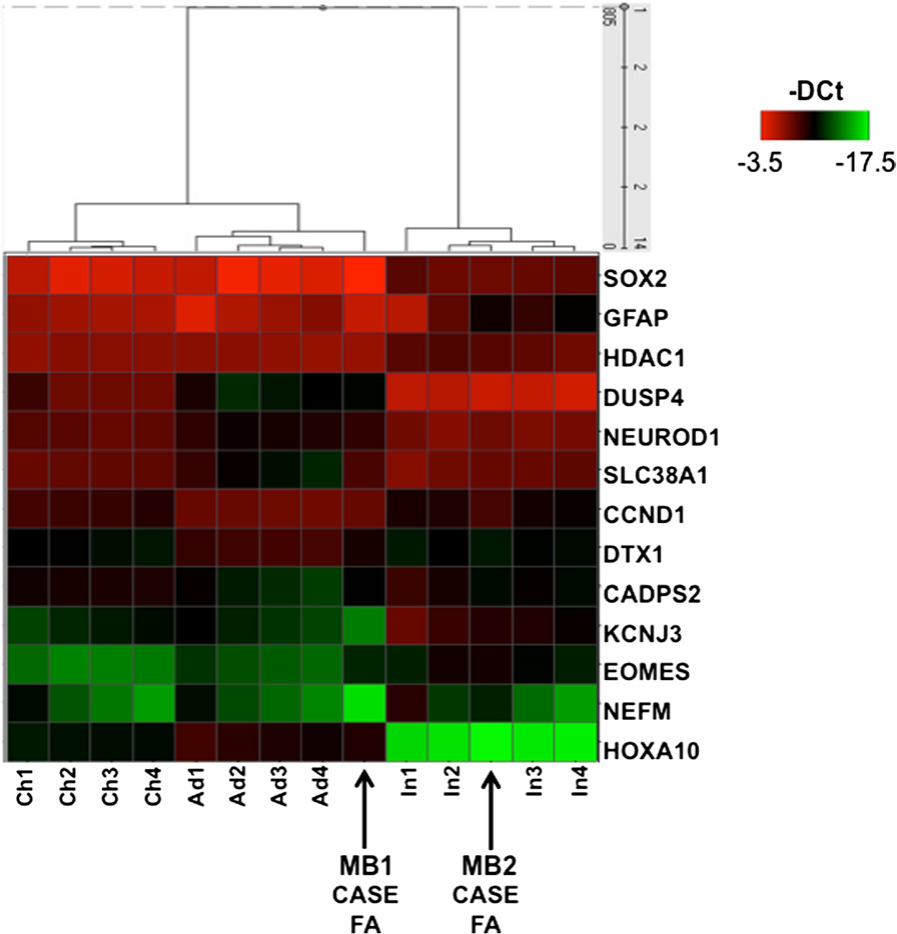
Heatmap and results of an unsupervised hierarchical clustering derived from the -DCt values of the analyzed genes in MB1 and MB2 (Case FA) and in adult (*n* = 4, A1, A2, A3, A4), childhood (*n* = 4, Ch1, Ch2, Ch3, Ch4) and infant (*n* = 4, I1, I2, I3, I4) SHH-MBs. Nodal numbers indicate bootstrap values obtained by resampling the data

Thus, we investigated the gene expression pattern of MB1 and MB2, compared to adult, childhood and infant sporadic SHH-MBs. We analyzed a panel of genes by RT-QPCR among the top 250 reported to be differentially expressed in the three classes of ages [43]. Our data are consistent with those reported by Kool 2014 that identified two major clusters mainly separating infant from childhood and adult SHH MBs. Moreover, our analysis showed that hemispheric MB1 (diagnosed under 3 years of age) clustered with adult/childhood MBs (Fig. 6), while vermian MB2 (diagnosed after 3 years of age) clustered with infant SHH MBs (Fig. 6).

### Discussion

This analysis allowed us to better define features of MBs in FA. We identified a new pathogenetic germline *BRCA2* mutation in FA; both MBs belonged to SHH molecular subgroup with a more “aggressive” phenotype in MB2, and, finally, we documented that MB1 shared similarities with sporadic adult SHH-MB, such as hemispheric localization and gene expression pattern.

In our patient, the first manifestation that led to the diagnosis of FA was the onset of WT.

WT and MB in FA have been reported in few cases and are associated with mutations of *FANCD1/BRCA2* and more rarely with mutations of *FANCN/PALB2* [24, 44]. Based on this evidence, we investigated *BRCA2* mutational status and confirmed the presence of biallelic truncating mutations in the FA patient. The c.658_659delGT mutation occurring on *BRCA2* exon 8 had been previously reported in hereditary breast/ovarian cancer cohorts (see in example [45]) and is one of the most frequently occurring in *FANCD1/BRCA2* mutated FA cases [29]. The second truncating mutation, the previously undescribed c.2944_2944delA (p.Ile982Tyrfs), occurs on *BRCA2* exon 11. The segregation analysis among the available relatives allowed us to assign the two mutations as of paternal and maternal origin, respectively. The absence of breast and ovarian cancer cases in the pedigree could appear surprising, at first. However, both paternal and maternal sides of the tree were characterized by a relatively small number of potential female carriers. Indeed, the c.658_659delGT allele was inherited by the patient via two male carriers (father and grandfather) and the only female member on this line (affected by gastric cancer) was not available for segregation analysis. The c.2944_2944delA allele, possibly coming from the mother’s maternal grandfather affected with pancreatic cancer, reached the patients via a 64 years old grandmother and a 36 years old mother, a female carrier of rather young age compared to 43 and 54 years, the mean ages of breast and ovarian cancer diagnosis, respectively, recently reported in a very large cohort analysis [46]. The preponderance of male relatives in this side of the tree further hindered any possible speculation on the role of the c.2944_2944delA on breast and ovarian cancer predisposition.

Complete *BRCA1* or *BRCA2* loss is lethal in mice [47]. This is likely to be the case also in humans, since no biallelic BRCA2 mutations occurring 5′ to exon 7 have been reported in FA patients so far, while monoallelic truncating mutations from exon 2 to exon 7 have been frequently associated with breast/ovarian cancer susceptibility. In three out of four FA patients, the c.658_659delGT mutation occurred in association with a truncating mutations of exon 11 [29]. This is also the case for the patient described here, in which the c.658_659delGT mutation is associated with a truncating mutation of *BRCA2* exon 11, the previously undescribed c.2944_2944delA (p.Ile982Tyrfs) mutation. Overall, these data suggest that BRCA2 truncation downstream of exon 7/8 is probably hypomorphic, but still compatible with life especially in association with exon 11 truncated BRCA2 forms. However, as already noticed by Meyer, homozygous or compound heterozygous exon 11 mutations have not been reported in FA patients despite the relatively high frequency of these alleles. Together with the report of the recurrent miscarriages in Jewish *BRCA2* mutation carriers [29], this suggests that biallelic exon 11 BRCA2 mutation may also be incompatible with life. More definitive answers on this topic may come from directly addressing this issue in specifically modeled mice.

MB in FA patients reported in the literature typically show cerebellar hemispheric localization and desmoplastic histology, as we observed in MB1 (Table 1). Cerebellar hemispheric MBs are frequently desmoplastic and belong to the SHH subgroup, especially in adults [48]. Hemispheric location is consistent with the results from MB mouse models that have shown SHH-MB origin from committed granule neuron precursors (GNPs) of the cerebellum. Teo et al., reported hemispheric involvement in 9 of 17 (53 %) SHH-MBs, regardless of age at diagnosis [49]. In Perreault study [48], SHH lesions rarely invaded the brain stem, consistent with a prior study that reported brain stem infiltration by WNT, but not by SHH tumors [50].

Gene expression analysis of our MBs allowed us to clearly define that both MB1 and MB2 belonged to SHH subgroup.

Biological explanation of the association between FA and SHH-MB has been analyzed in different papers. Frappart et al. first showed that *BRCA2* loss affects neurogenesis and promotes MB growth, using a mouse model harboring neural tissue-restricted *BRCA2* inactivation [51]. They also identified *PTCH1* (a gatekeeper gene often inactivated in MB) as a critical target in all DNA repair-deficient MB.

**Fig. 7:**
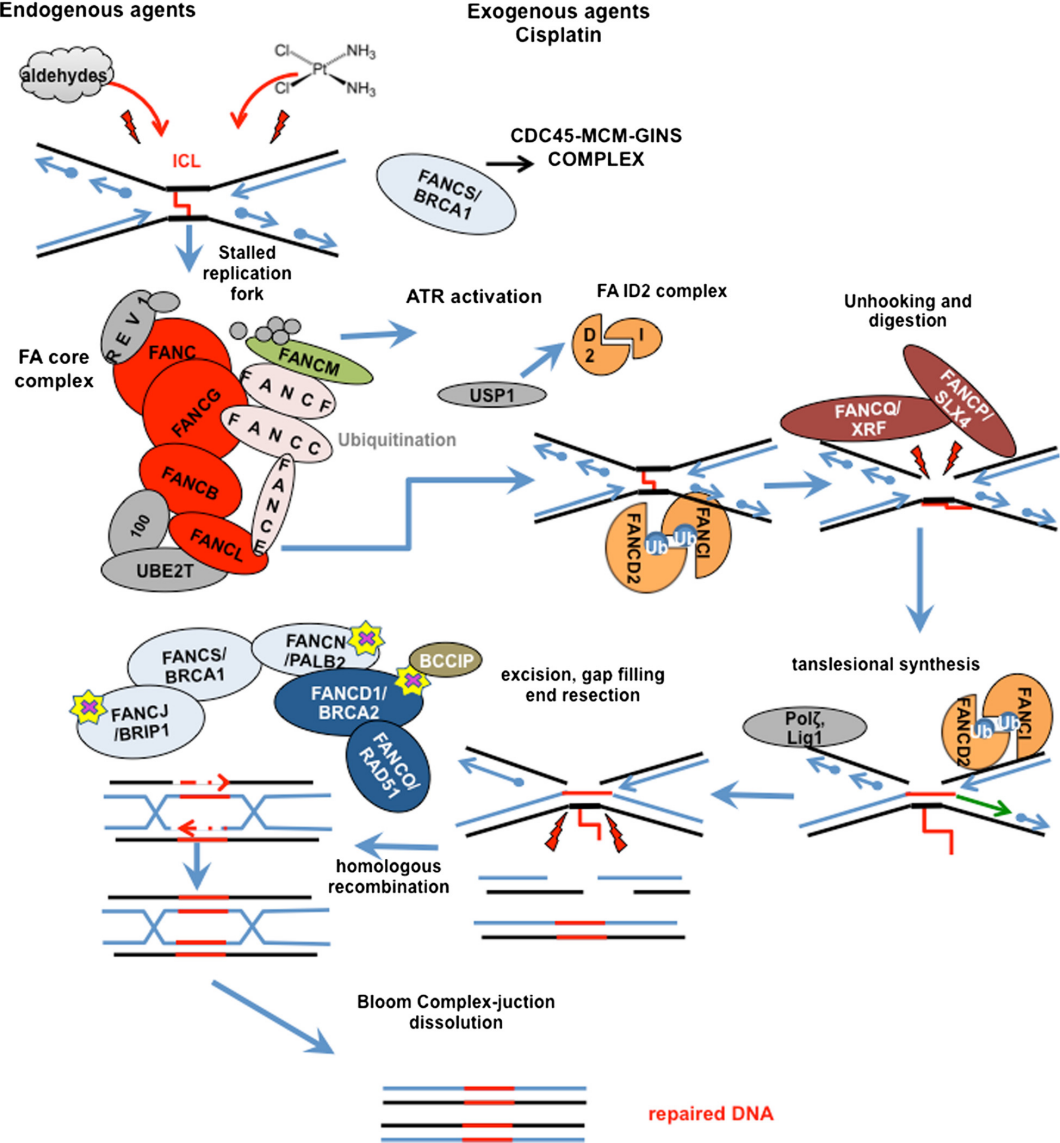
Fanconi Anemia Pathway. Endogenous and exogenous agent cause interstrand crosslink lesions (ICL). When an ICL occurs, the replication fork is stalled and the FA pathway is activated with the recruitment of the FA core complex (FANCA, B, C, E, F, G, L, and M). FANCL ubiqiuitinates the FANCD2-FANCI (ID2) complex that guides the nucleolytic incisions and translesion synthesis repair events by “unhooking” the ICL. This allows the homologous recombination repair through FANCD1/BRCA2, FANCJ/BRIP1, FANCN/PALB2, FANCO/RAD51C, and FANCS/BRCA1. Yellow stars highlight FA/HR repair molecules that have been found genetically inactivated in FA patients with medulloblastoma. BCCIP is a BRCA2 interacting protein which is able to induce medulloblastoma growth when genetically loss in concomitance with p53 deletion (See text). Readapted from [52]

Indeed, defective DNA repair mechanisms render FA cells prone to DNA interstrand crosslinks (ICLs) caused by both endogenous (e.g. reactive aldehydes) and exogenous agents (e.g. alkylating chemotherapeutic drugs) [1, 5, 52, 53]. The FA pathway is activated by an ICL during the S phase: the replication fork is stalled and FA core complex is recruited (FANCA, B, C, E, F, G, L, and M). Then, FANCL ubiquitinates the FANCD2-FANCI (ID2) complex, essential for nucleolytic incisions and translesion synthesis repair events. The complex then “unhooks” ICL, allowing homologous recombination repair by FANCD1/BRCA2, FANCJ/BRIP1, FANCN/PALB2, FANCO/RAD51C, and FANCS/BRCA1 (Fig. 7).

Huang et al. reported that conditional knockdown *BCCIP* (BRCA2-interacting protein with a role in homologous recombination and chromosome stability) in concomitance with p53 deletion caused rapid development of MB in the external granular layer, triggered by SHH pathway activation [54] *via* the inactivation of the Ptch1 gatekeeper tumor suppressor [54]. As reported above, biallelic *PALB2/FANCN* mutation carriers show a cancer spectrum comparable to those with biallelic *BRCA2/FANCD1*, characterized by early onset AML and embryonic tumors, including MB and WT [44]. This observation strengthens the role of BRCA2 in medulloblastomagenesis, since FANCN (also known as ‘partner and localizer of BRCA2′, PALB2) is well known to co-localize with BRCA2 in the nucleus [55]. Interestingly, *BRIP1/FANCJ* maps on 17q chromosome, a region frequently deleted in MBs [56]. Thus, deleterious mutations in one of the members of the homologous recombination machinery could lead to MB onset (Fig. 7). This feature may provide the rationale for new therapeutic approaches for MB, as also suggested by Bayrakli et al. [26]. Platinum compounds or similar drugs, inducing growth arrest by ICL-stalled- DNA replication fork, could be combined with agents which take advantage of the incapacity of HR-deficient neoplastic cells to face up this stall (e.g. enzyme poly-ADP-ribose polymerase I) [26]. Indeed, PARP- inhibition has already been shown to sensitize childhood glioma, medulloblastoma and ependymoma to radiation [57].

It has been reported that MBs do not change subgroup at the time of recurrence and that SHH tumors more frequently have tumor bed recurrence than metastatic dissemination [58–60]. Even though we cannot completely rule out that the second MB was not a distant relapse from the first one, in our patient, the occurrence of MB2 with a different localization, a more aggressive histology, and distinct gene expression patterns could be suggestive for a second tumor. We hypothesize that Hedgehog signaling activation could have been triggered in two distinct cells of origin. Indeed, MBs have long been known to arise in GNPs in the external granule layer (EGL) and, more recently, GNPs derived from cochlear nuclei of the brainstem [61]. SHH-dependent MBs may also arise from neural stem cells (NSCs) of the subventricular zone (SVZ). The different tumor location and expression levels of stemness/differentiation genes sustain this assumption. MB2 was characterized by higher levels of EZH2, which trimethylates histone 3 lysine 27 (H3K27me3), concurrently with down regulation of the lysine demethylase 6A (KDM6A), which removes the same repressive mark on chromatin (Fig. 5e). Both these events converge on shutting off oncosuppressor/differentiation genes and contribute to the maintenance of a more “cancer stem-like” phenotype [37, 62].

It has been reported that pediatric and adult SHH-MBs are clinically and molecularly distinct [42, 43]. In a data analysis based on Whole Genome Sequencing Kool et al. reported that the molecular differences among infant, childhood and adult SHH-MBs reside not only at the transcriptional level, but also in DNA methylation [43].

In our report, we found that the hemispheric, desmoplastic, myc-not amplified SHH MB1 was more similar to other adult/childhood SHH-MBs, while the vermian, large cell/anaplastic, N-myc amplified MB2 was more similar to infant SHH-MB. The hypothesis of different cells of origin stands also for such differences between adults and infants SHH-MBs.

Lastly, the suspect of SHH-MB in FA could direct the therapy toward a tailored approach, based on the use of PARP inhibitors or/and SHH inhibitors. However, giving the specific genetic background (*BRCA2*), it is highly unlikely that these tumors have *PTCH1* or *SMO* mutations making them sensitive to the ‘upstream’ Hedgehog inhibitors targeting SMO. It is more likely that they have activated the SHH pathway more downstream at the level of *MYCN* and probably also *GLI2* amplification (as probably in our MB2). Indeed, as Kool et al. have shown in their recent paper, these tumors are resistant to these ‘upstream’ Hedgehog inhibitors [43]. Inhibitors that target downstream components of the Hh pathway are under pre-clinical evaluation [63] (e.g. Gant61 [64], Glabrescione B [65]).

## Conclusion

In conclusion, we report a novel *BRCA2* mutation in a FA patient with diagnosis of two distinct MBs. Molecular features of MB in our *FANCD1/BRCA2* patient highlight that MB in FA patients belongs to SHH subgroup.

Two points are worthy of discussion, due to potential clinical implications. First, it is important to suspect a diagnosis of FA in patients whose first manifestations are embryonic tumors with a sequence WT-MB.

Second, the identification of SHH subtype MB in FA patients with *FANCD1/BRCA2* mutations may play a critical role for targeted therapeutic interventions. Indeed, in patients characterized by a peculiar difficulty in cancer therapeutic management, due to the underlying chromosomal instability, a benefit could be provided by target therapy or new combined therapies.

### Consent

Written informed consent was obtained from the patient’s parents for publication of this Case report and of any accompanying images. A copy of the written consent is available for review by the Editor-in-Chief of this journal.

## Additional files

Additional file 1: Figure S1. Imaging of WT. CT scan: two-dimensional coronal post-contrast CT reconstruction. The image (**a**) shows a large mass (arrows) arising from the fused pelvic kidney (cake kidney). The mass is multi-lobulated and heterogeneous in attenuation; there are many lung metastases (**b**). CT scan after neoadjuvant chemotherapy (**c**) showing a decrease in tumor size and central necrotic changes. Additional file 2: Figure S2. Molecular characterization of MB1 and MB2. Heatmap showing mRNA levels of the indicated genes in MB1, MB2 and normal cerebella as control (CTR). Genes are grouped depending on the molecular subgroups which they identify (SHH, WNT, GROUP 3, GROUP 4). A green-red color scale depicts normalized Delta Ct values (green, lower expression, red, higher expression).

## Abbreviations

FA: Fanconi Anemia
MB: Medulloblastoma
SHH: Sonic Hedgehog
WT: Wilms Tumor
AML: Acute myeloid leukemia
MDS: Myelodysplastic syndrome
SIOP: International Society of Paediatric Oncology
FISH: Fluorescent in situ hybridization
CT: Computed Tomography
MRI: Magnetic Resonance Imaging
DEB: Diepoxybutane
WHO: World Heath Organization
GNPs: Granule neuron precursors
EGL: External granule layer
NSCs: Neural stem cells
SVZ: Subventricular zone
