# Supplementary figures and images for: Characterization of medulloblastoma in Fanconi Anemia: a novel mutation in the BRCA2 gene and SHH molecular subgroup
Source: Biomark Res. 2015 Jun 6;3:13. doi: 10.1186/s40364-015-0038-z (PMC4462002; doi:10.1186/s40364-015-0038-z)

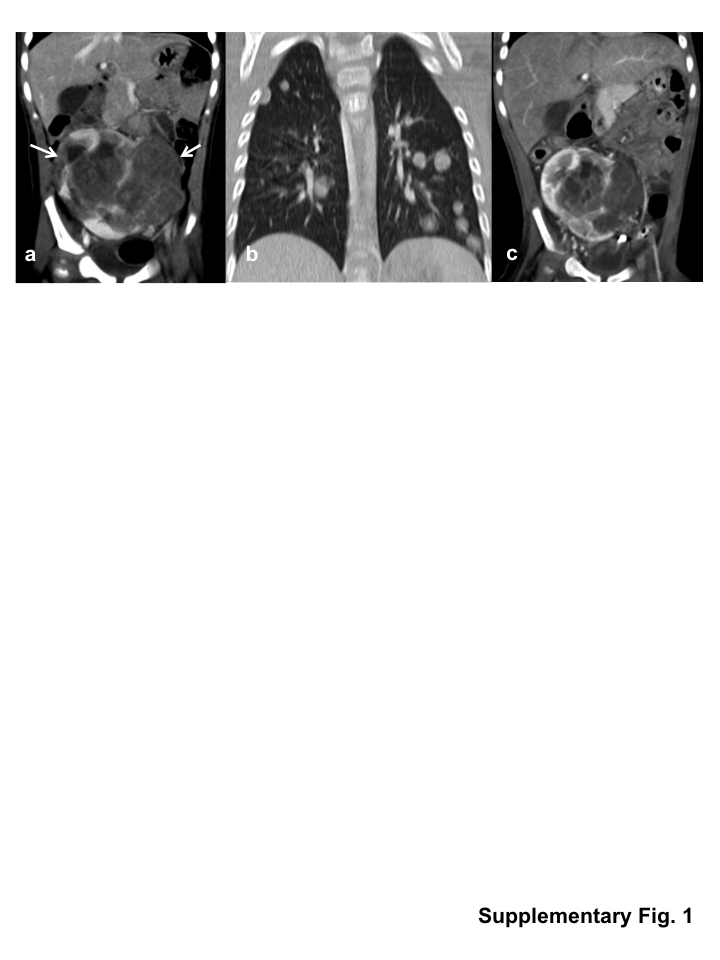

Supplement: Additional file 1: Figure S1. — Imaging of WT. CT scan: two-dimensional coronal post-contrast CT reconstruction. The image (a) shows a large mass (arrows) arising from the fused pelvic kidney (cake kidney). The mass is multi-lobulated and heterogeneous in attenuation; there are many lung metastases (b). CT scan after neoadjuvant chemotherapy (c) showing a decrease in tumor size and central necrotic changes. [file 40364_2015_38_MOESM1_ESM.tiff]

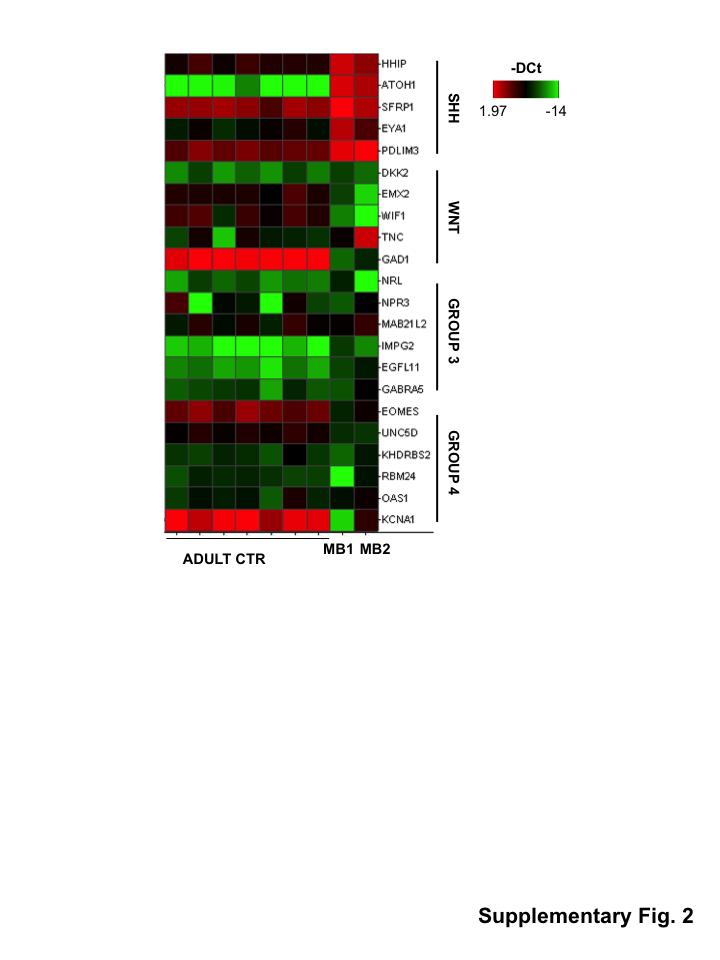

Supplement: Additional file 2: Figure S2. — Molecular characterization of MB1 and MB2. Heatmap showing mRNA levels of the indicated genes in MB1, MB2 and normal cerebella as control (CTR). Genes are grouped depending on the molecular subgroups which they identify (SHH, WNT, GROUP 3, GROUP 4). A green-red color scale depicts normalized Delta Ct values (green, lower expression, red, higher expression). [file 40364_2015_38_MOESM2_ESM.tiff]
